# Identification and preliminary characterization of *Plasmodium falciparum* proteins secreted upon gamete formation

**DOI:** 10.1038/s41598-022-13415-7

**Published:** 2022-06-10

**Authors:** Felicia Grasso, Federica Fratini, Tanino Giuseppe Albanese, Stefania Mochi, Mariagrazia Ciardo, Tomasino Pace, Marta Ponzi, Elisabetta Pizzi, Anna Olivieri

**Affiliations:** 1 Dipartimento Di Malattie Infettive, Istituto Superiore Di Sanità, Rome, Italy; 2 Servizio Grandi Strumentazioni E Core Facilities, Istituto Superiore Di Sanità, Rome, Italy

**Keywords:** Parasite biology, Parasite physiology

## Abstract

Malaria long-term elimination depends on parasite transmission control. *Plasmodium* sexual stage maturation in the mosquito, including egress from the host erythrocyte, is one of the prime targets for transmission-blocking interventions. This work aims to identify candidate molecules potentially involved in gamete emergence from the host erythrocyte, as novel transmission blocking targets. We analyzed by quantitative mass spectrometry the proteins released/secreted by purified *Plasmodium falciparum* gametocytes upon induction of gametogenesis. The proteome obtained showed a good overlap (74%) with the one previously characterized in similar conditions from gametocytes of the rodent malaria parasite *P. berghei*. Four candidates were selected based on comparative analysis of their abundance values in released vs total gametocyte proteome. We also characterized the *P. falciparum* orthologue of the microgamete surface protein (MiGS), a marker of male gametocyte secretory vesicles in murine models of malaria. The findings of this study reveal that all the selected candidate proteins are expressed in both genders and localize to vesicle-like structures that respond to gametogenesis stimuli. This result, together with the fact that the selected proteins are released during gamete emergence in both *Plasmodium* species, makes them interesting candidates for future functional studies to investigate their potential role in the gametogenesis process.

## Introduction

Malaria is a devastating disease, with 229 million cases and 409 thousand lethal outcomes in 2019^1^. The burden is heaviest in Africa, where more than 90% of all malaria deaths occur, mostly in children under 5 years of age. The parasite *Plasmodium falciparum* is the deadliest among the species that affect humans, being responsible for the vast majority of lethal events.

*Plasmodium* asexual stages are responsible for the clinical manifestations of the disease, while transmission relies on the sexual stages, called gametocytes. When gametocytes are ingested by mosquitoes, they are induced to differentiate into gametes by the temperature drop and the presence of xanthurenic acid in the mosquito midgut^2^. Each female gametocyte forms a single macrogamete, while male gametocytes undergo a drastic transformation, known as exflagellation, and form eight flagellated microgametes. For mating to occur, gametes must egress from the host erythrocyte. This process takes place by successive inside-out rupture of the two membranes surrounding the parasite, the parasitophorous vacuole membrane and the host cell membrane^3^. A few minutes before egressing, some female gametocyte secretory organelles, the osmiophilic bodies (OBs), migrate to the cell periphery and release their content in the parasitophorous vacuole lumen. Concomitantly, the parasitophorous vacuole membrane (PVM) disintegrates at multiple sites and erythrocyte membrane ruptures by the formation of a single pore about 15 min after the blood meal^4–6^. A male-specific type of secretory vesicles, Male Osmophilic Bodies (MOBs), was shown to be involved in PVM rupture of *P. berghei* male gametocytes upon induction. MOBs share part of their proteome with female OBs, while differ in their shape and mechanism of discharge^7^. In *P. falciparum* male gametocytes, these male-specific vesicles were not identified^8^.

Once free from the host cell, male and female gametes fuse to form the fertilized zygote. Over the ensuing 24–36 h, the zygote transforms into a motile ookinete, which crosses the midgut epithelium to form an oocyst, where new infectious parasites are generated.

The gamete formation process leads to an approximate 300 fold loss of parasite abundance, representing a bottleneck in the parasite life cycle^9^. In this context, even a slight reduction in efficiency may dramatically affect parasite survival. The midgut stages in general and in particular egress from the host cell are thus viewed as prime targets for transmission-blocking interventions.

Previous work from the authors led to the identification of the proteins released during *P. berghei* gamete development^10^. By a proteomic approach, here we compiled a list of proteins released upon *P. falciparum* gamete formation and investigated the subcellular localization before and after gametocyte activation of selected candidates.

## Methods

### Parasite culture and gamete activation

*P. falciparum* 3D7 line, cultured in human 0+ erythrocytes according to standard methods^11^, was induced to produce gametocytes by starvation and asexual stages were killed 24 h after induction by 0.05 M N-acetyl glucosamine treatment, to obtain synchronous gametocytes. At day five post induction, immature stage III *P. falciparum* gametocytes were purified by 60% Percoll gradient to remove uninfected erythrocytes and put back into culture for additional 5 days (Fig. S1). Mature gametocytes were then activated to form gametes, by exposing them to activation medium (RPMI with gentamicin at room temperature, pH 8.2) for 20 min. Parasites were removed by centrifugation followed by filtration and supernatants were collected with addition of protease inhibitors (Roche).

### Proteomic analysis

Protein mixture (ca. 125 μg) derived from 1 ml of gametocyte egress supernatant (Experiment 1), was divided in three samples, to perform technical replicates (R1-3) that were then resolved by SDS-PAGE on 4–12% precast minigels (NuPAGE Novex Bis–Tris; Invitrogen). As previously described^12^, following staining with NuPage Colloidal Coomassie (Invitrogen), gel lanes were cut into 10 slices for in-gel tryptic digestion. In a second biological replicate (Experiment 2), 24 slices were cut and digested. Nano-RPLC was performed using a nano-HPLC 3000 Ultimate (Dionex) connected in line to LTQ-XL linear ion trap (Thermo Fisher) as previously described^10^. Briefly, tryptic digests were packed on a C18 RP-precolumn (300 µm i.d. × 5 mm; 5 μm particle size; 100 Å pore size; LC Packings-Dionex) and then resolved on a homemade 12 cm × 75 µm- i.d. Silica PicoTip (8 ± 1 µm) column (PicoTip Emitter, NewObjective) packed with Magic C18AQ resin (5 μm particle size; 200 Å pore size, Michrom Bioresouces Inc.) for chromatographic separations. Peptides were eluted at 0.3 µL/min along a 60 min linear gradient from 15 to 60% of buffer B (95% ACN, 0.1% FA) and electrosprayed directly into the mass spectrometer with a spray voltage of 1.60–1.65 kV and a capillary temperature of 180 °C^10^. Data acquisition was performed in data-dependent Top5, with a maximum injection time of 100 ms; *m/z* 50–2000 mass range; minimum signal threshold of 200 counts; isolation width of 2; normalized collision energy of 35. Wideband and multistage activation were enabled. The dynamic exclusion was enabled with a repeat count of 2 within 30 s and exclusion time of 60 s.

Spectra files (available at ftp://massive.ucsd.edu/MSV) were analyzed by Sequest HT search engine with Proteome Discoverer 1.4 (ThermoFisher) using a homemade database constructed with the Human Uniprot-Swissprot reviewe database (released on June 2020) and Pfalciparum3D7_version46 of Plasmodb. The search was run also again the decoy database. The Carboamidomethylation of cysteines was specified as fixed modification and the oxidation of methionine was set as variable modification; mass tolerance was set to 1 Da for precursor ion and 0.4 Da for fragment ions and a maximum of two missed cleavages was allowed. The Percolator tool was used for peptide validation based on the q-value and high confidence was chosen, corresponding to a false discovery rate (FDR) ≤ 1% on peptide-level. Proteins were identified with a minimum of 2 peptides rank = 1. Protein abundance values were determined by Top3 method^13^ considering the three (or two) most abundant unique peptides for each protein.

### Statistical analysis

The proteome obtained by proteomic analysis (technical replicates R1-R3) was compared with a previously published proteomic dataset from non-induced gametocytes, (available in three biological replicates: G1–G3)^14^ to define proteins over or down represented. Protein abundance values were normalized on the replicate with the highest overall abundance value (calculated as the sum of values assigned to each identified protein). The normal distributions of normalized Top3 values between the technical replicates R1-R3 was verified by Kolmogorov–Smirnov test (alpha = 0.05), while the reproducibility was assessed by two-tailed t-test (P > 0.5; alpha = 0.01; DF = 93) to confirm the equality between means and by linear regression analysis (ANOVA P < 10–3; alpha = 0.05; DF = 91).

The distribution of the log2 ratio between the means of R1-R3 and G1-G3 was fit by a Gaussian function (Kolmogorov–Smirnov test; alpha = 0.05). Linear regression analyses were conducted by ANOVA (P < 10–3; alpha = 0.05).

Statistical analyses were performed by XLSTAT 2020.1.1 (Addinsoft (2022), XLSTAT statistical and data analysis solution. New York, USA. https://www.xlstat.com).

### Western blot analysis

Protein extracts from *P. falciparum* stage V gametocytes and non-infected human ghosts obtained by hypotonic lysis^15^ as negative controls were separated on 12% SDS polyacrylamide gel and transferred to a nitrocellulose membrane (GE Healthcare) using MINI TRANS-BLOT® (Biorad). After blocking overnight with 5% nonfat dry milk in PBST (PBS with 0.1% Tween 20), membranes were incubated with primary specific antibodies (1:1000 dilution), followed by incubation with anti-mouse horseradish peroxidase-conjugated secondary antibody (1:10,000 dilution). The immunocomplexes were visualized using chemiluminescence ECL detection system (Luminata Forte Western HRP Substrate, Millipore) according to manufacturer's instructions.

### Immuno-fluorescence assays

As previously described^16^, blood smears from parasite cultures were fixed in 4% paraformaldehyde for 30 min at room temperature, permeabilized with 0,1% Triton-X100 in PBS for 10 min and incubated for 1 h with the primary antibodies at a 1:100 dilution (except anti-Pfg377 and anti-Tubulin used at 1:400 dilution), followed by secondary antibodies: anti-mouse and anti-rabbit fluorescein (Invitrogen and ThermoFisher respectively) 1:200 dilution, anti-mouse and anti-rabbit rhodamine (ThermoFisher) 1:200 dilution and the nuclear marker DAPI (Life Technologies) 500 ng/ml. After washing, smears were mounted in Vectashield (Vector Laboratories). Negative controls without primary antibodies have been performed, resulting in complete absence of fluorescence signals. At least 200 cells were observed in each immuno-fluorescence assay. The percentage of overlap between green and red fluorescence was determined using ImageJ software on four independent immunolocalization images per antibody used.

### Ethics approval

Blood for analyzing erythrocyte proteins and for propagating *P. falciparum* cultures was obtained from the Transfusion Center of Policlinico Umberto I. The experimental protocol was approved by the “Policlinico Umberto I Ethics Committee”. All methods were performed in accordance with relevant guidelines and regulations on suitability assessment of blood donors and blood components (Ministry of Health-Decree of 3 march 2005, Official Gazette no 85, 13-4-2005). Blood samples were screened for known pathogens in accordance with the Italian National Regulations. No information about the donor, other than the blood group was obtained or recorded by the user. A written informed consent was asked to blood donors, including a statement that participation was voluntary. No minors were included in this study.

## Results and discussion

**Figure 1:**
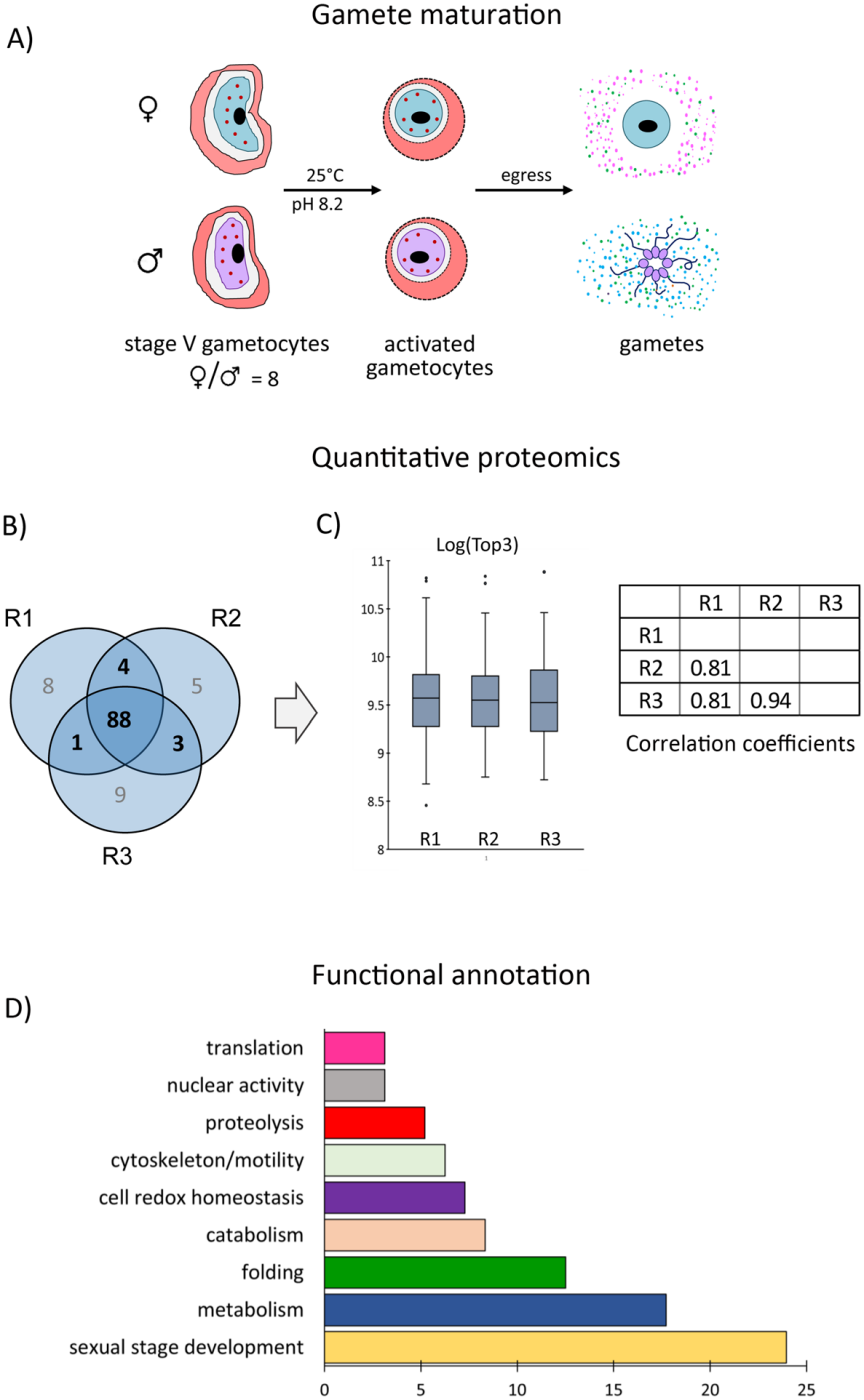
(**A**) Experimental design for characterization of proteins released by activated *P. falciparum* gametocytes. Enriched stage V gametocytes were induced to form gametes by a drop in temperature in induction medium. Culture supernatants were collected by centrifugation and proteins released during gametogenesis were aceton-precipitated and subjected to mass spectrometry analysis. (**B**) the overlap between the proteins identified by mass spectrometry in each technical replicate (R1–R3) is shown as a Venn diagram; (**C**) the reproducibility assessment of proteomic data is schematized. The distributions of Top3 values of proteins identified in 2 out of 3 replicates (R1–R3) are represented as box plots (T-test probabilities P = 0.94). Pearson’s correlation values calculated for each pair of replicates are reported in the Table (ANOVA P < 10–3). (**D**) Functional analysis based on GO annotations available in PlasmoDB is shown as a histogram.

Purified mature stage V gametocytes were induced to form gametes, by exposing them to induction medium for 20 min at 25 °C (Fig. 1A). After parasite removal, supernatants were collected for proteomic analysis. We also attempted to produce supernatants from non-induced gametocytes as a control, but we could not achieve this result, since in all preparations a relevant fraction of gametocytes got induced by the unavoidable centrifugations and temperature drop even if not exposed to induction medium.

Mass spectrometry analysis was then performed on proteins released upon induction of gametogenesis (experiment 1) in three technical replicates (R1–R3) with an overlap of 87% (Fig. 1B and Supplementary Table S1). We then considered for further analysis 96 proteins, identified in at least two replicates. Proteins from this dataset exhibit an 87.5% overlap with a second biological replicate (Experiment 2) performed to confirm robustness of the identified proteome (supplementary Table S1).

The majority of the 96 proteins dataset are more expressed in female gametocytes than in males as resulted by comparative analysis with a previously published gender-related *P. falciparum* gametocyte proteome^17^, (Supplementary Table S2). This is explained by the fact that, in the culturing conditions used, the sex ratio of the 3D7 strain is usually 1:10 males to females. We thus expected to identify mainly proteins released either by female gametocytes or by both genders.

Of the 96 proteins considered, 89 have an orthologue in *P. berghei*. Of these, 66 (corresponding to 74%) had been identified in our previous analysis of proteins released upon gamete induction in *P. berghei*^10^, indicating a good conservation degree of gametocyte secretomes in the two species (Supplementary Table S2).

We also compared the relative abundances of the 96 proteins identified in experiment 1 (from now on defined as R dataset) with those of the same proteins belonging to a previous published proteomic dataset from non-induced total mature gametocytes (G dataset)^14^. The rationale behind this comparison is that relative abundance of proteins actively secreted during gametogenesis is expected to be similar to that observed in non-induced gametocytes, where the same proteins are still retained inside the cell. Proteins accumulated in late gametocytogenesis or re-expressed upon induction should be, instead, over-represented in the egress supernatant. This comparison would also enable us to distinguish contaminant proteins due to ruptured cells, expected to be less represented in the egress supernatant than in total gametocytes.

Each protein abundance in the two datasets was normalized on the replicate with the highest total abundance value (Supplementary Table S2). The good reproducibility of replicates R1-R3 was verified after normalization by T-test (P ≥ 0.5) and linear regression (R ≥ 0.8; P ≤ 10–3) (Fig. 1C) and the mean abundance values of R and G datasets were used for successive analyses.

**Figure 2:**
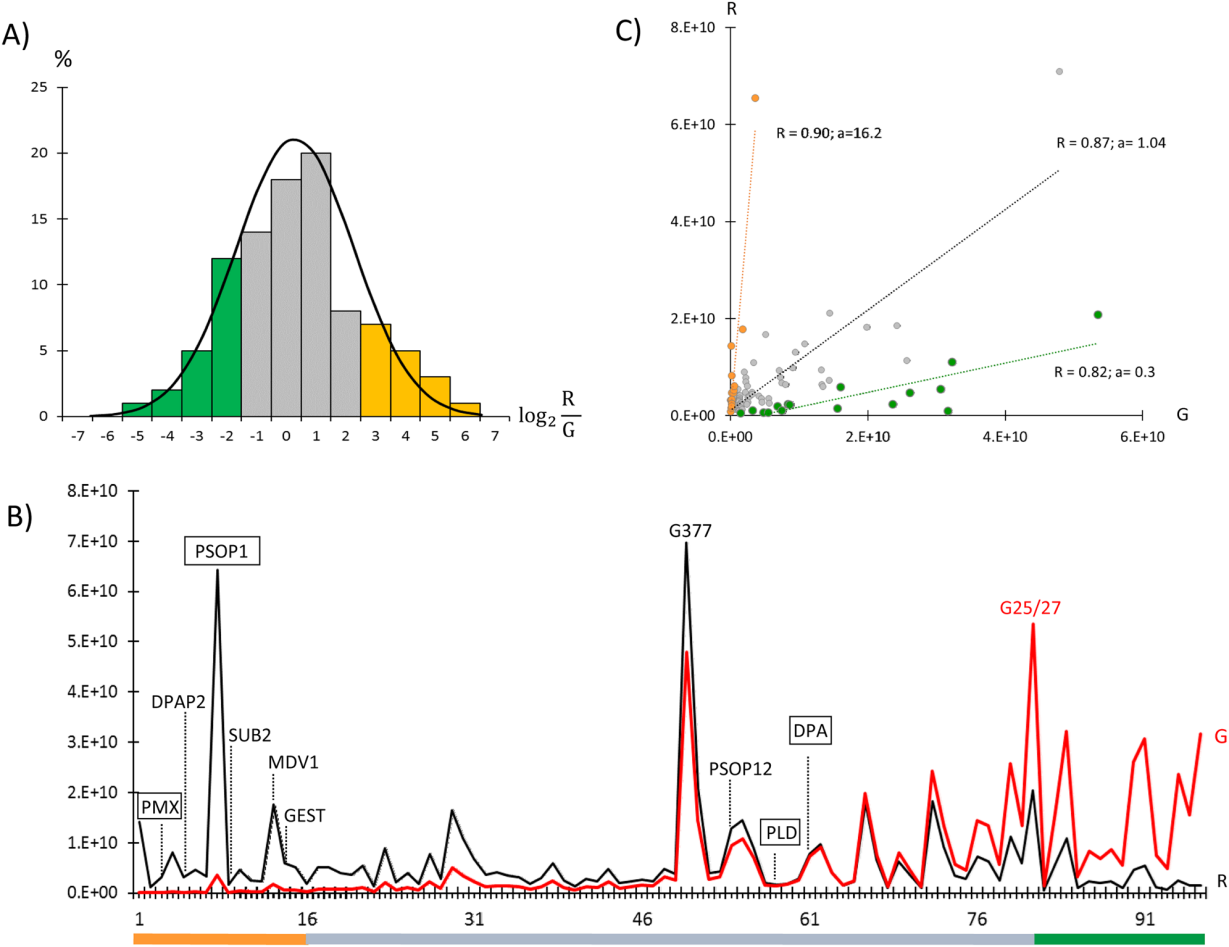
Statistical analysis of the proteome released by activated gametocytes. (**A**) The log2 distribution of the ratio between the mean abundance values of released proteome R and those of the same protein dataset in total gametocytes G (R/G) is shown as a bar chart; black line represents the corresponding fit Gaussian function. Orange and green bars highlight values exceeding the mean (m = 0.8) plus and minus one standard deviation (sd = 2.1) respectively. (**B**) G (red line) and R (black line) abundance profiles are ranked according to descending log2 ratio values. The orange bar below the plot corresponds to Top3 ratio values higher than the mean of at least one standard deviation; the green bar to ratio values lower than the mean of at least one standard deviation; the grey bar to values around the mean. Abundances of known egress markers (DPAP2, SUB2, MDV1, GEST G377 and PSOP12), selected egress-related candidates (boxed) and the contaminant G25/27 are indicated. (**C**) Plot of R vs G highlights protein abundance values around the mean (gray dots) and, values exceeding one standard deviation from the mean (orange and green dots). The correlation values R (ANOVA P ≤ 10–3, alpha = 0.05) and the linear coefficient (a) of the regression lines (dotted) are indicated.

The comparison, performed on the log2 distribution of R/G ratios, showed that out of the 96 proteins considered 16 have ratio values higher and 16 lower than one standard deviation from the mean value of the distribution (Fig. 2A and Table S2). The presence of three distinct groups of proteins was also confirmed by ranking the abundance values of R and G datasets according to their ratio values (Fig. 2B). We, in fact, observed proteins abundant in secreted/released proteome (R) but poorly represented in total non-induced gametocytes (G) and vice versa, as well as proteins with comparable abundance values in the two dataset. Within the three groups, proportionality of relative abundances is maintained between R and G, as confirmed by the linear regression analysis (R ≥ 0.8) (Fig. 2C).

Proteins less abundant in the gametogenesis secretome compared to total gametocyte proteome include, as expected, proteins non-specifically involved in the egress process. Among them the cytoplasmic, gametocyte-specific G25/27 (see Fig. 2B), the most abundant protein in the whole gametocyte proteome^14^ and the group of adhesive proteins CCp (Table S2). These latter localize to the PV lumen in non-activated gametocytes, while forming a large complex with Pfs230 at the surface of female gametes^18^.

We then looked at the R/G ratios of proteins known to participate in the egress process. The OB-resident PfG377, a structural protein involved in the formation and shaping of the OBs^19^ has relative abundance values similar in the two datasets (Fig. 2B and Table S2) but, interestingly, most of the egress-related markers fall in a protein group more abundant in the proteome of released proteins. These include most of the proteins known to localize to OBs: the gamete egress and sporozoite traversal protein (GEST), the dipeptidyl aminopeptidase 2 (DPAP2), the subtilisin-like protease 2 (SUB2)^14^ and the male development gene 1 (MDV1) localizing to the gametocyte parasitophorous vacuole and released during gamete emergence (ref) (Fig. 2B and Table S2).

Between the proteins identified, we selected for further investigation four proteins, based on their R/G ratio (Fig. 2B): the secreted ookinete protein 1 (PSOP1, PF3D7_0721700)^10^ and the Plasmepsin X (PMX, PF3D7_0808200)^20^ with a ratio value higher than one standard deviation from the mean (Table S2); a putative lactate dehydrogenase (PLD, PF3D7_1325200), and a putative deoxyribose-phosphate aldolase (DPA, PF3D7_1021600) with ratio values around the mean^10,12^. All the selected proteins were detected also in the proteome released by *P. berghei* gametocytes during gametogenesis and shown to localize to gametocyte secretory vesicles^10^.

Specific antibodies raised against conserved regions of these proteins^10,12^ were tested on *P. falciparum* lysates and uninfected human ghost preparations to confirm their specificity also in this species (Fig. S2 and S4).

We also wanted to confirm for two selected egress-related proteins, PSOP1 and GEST, the predicted differences in relative abundance (Fig. 2) between released proteins and non-induced gametocytes by western blot analysis. As shown in Fig. S3, the PSOP1-specific signal was detected only in the supernatant of gametogenesis but not in the total extract of non-induced gametocytes, where this protein was likely under the detection limit. GEST was, instead, detected in both samples but with a higher intensity in the released proteins. This result supports the idea that a number of egress-related proteins may be accumulated in fully mature gametocytes or re-expressed upon induction.

**Figure 3:**
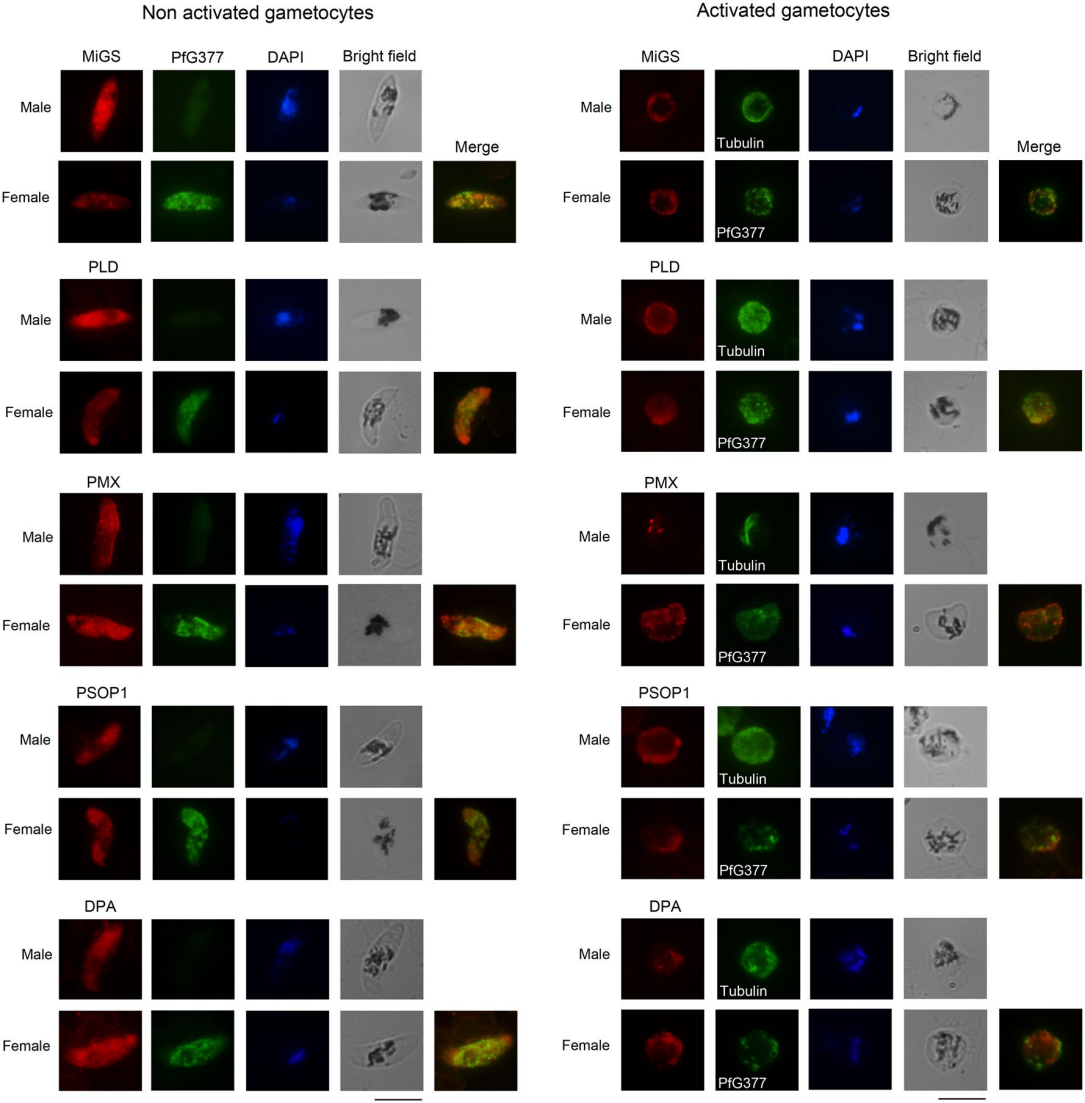
Subcellular localization before and after gametocyte activation. Immuno-localization of mature *P. falciparum* gametocytes before and after activation, by using specific immune sera against four proteins selected from the activated gametocytes secretome and PfMiGS, ortholog of a murine male-specific protein. Pfg377, a female-specific OB marker and alpha-Tubulin, staining male gamete forming flagella, were used to distinguish between genders.

We then investigated the subcellular localization of the four selected proteins in immuno-fluorescence assays (IFAs). In *P. falciparum* gametocytes, they localize to punctuate structures and show poor colocalization with the OB marker Pfg377 (Fig. 3) that never exceeds 15% of total fluorescence (Table S3). This suggests that also in this species the gametocyte egress-related secretome includes proteins localizing to vesicle-like structures distinct from OBs. 5–8 min after induction to form gametes, all the four proteins moved to cell periphery, suggesting that different classes of gametocyte cytoplasmic vesicles respond to gametogenesis induction stimuli (Fig. 3).

This is the first report that identifies proteins secreted/released by activated *P. falciparum* gametocytes and defines their relative abundance compared to non-activated gametocytes. Our study also suggests that a fraction of egress-related molecules is progressively accumulated during the final steps of maturation or re-expressed upon induction of gametogenesis.

Four candidate proteins localized to non-OB vesicles. Unfortunately, we could not establish whether these proteins identify the same or distinct classes of vesicles, as we could not perform co-localization experiments between them, being all antisera raised in mice.

Two of the selected candidates, PMX and PLD, were described as male-specific in *P. berghei*^10^. However, in a previous study, it had been proposed that PMX may be involved in egress of gametocytes of both genders from the host cell^21^, thus suggesting that a low-level expression of PMX is likely to occur also in female gametocytes. The other two candidates, PSOP1 and DPA, were detected both in male and female *P. berghei* gametocytes^10,12^. In *P. falciparum*, the four proteins were expressed in both genders. Only PLD gave a stronger signal in male gametocytes compared to females, consistent with previous proteomic reports^17^. PSOP1, DPA and PMX showed a dotted pattern in both genders, similarly to what observed in *P. berghei*^10^, while PLD showed a punctate pattern in female gametocytes but appeared diffused in the cytoplasm in male gametocytes. The detection of PMX in gametocytes both by proteomics and immunolocalization, contradicts previous reports that identified the protease in gametes and ookinetes, but not in gametocytes^22^.

This is one of the first reports of secretory vesicles in *P. falciparum* male gametocytes, where neither MOB-like nor other secretory vesicles were identified by electron microscopy^8^.

With the aim of investigating whether OB-like structures existed in *P. falciparum* male gametocytes, we characterized by IFA the subcellular localization of the microgamete surface protein (MiGS, PF3D7_1234400), a male-specific putative aspartyl protease localizing in *P. berghei* MOBs, but not identified in our female-biased gametocyte secretome. Our results showed that in *P. falciparum* MiGS is expressed in both genders, but upregulated in male gametocytes, consistently with previous proteomic reports^17^. In both female and male gametocytes, the protein showed a punctate signal, moving to cell periphery in activated gametocytes. This result suggests that also *P. falciparum* male gametocytes have secretory organelles responsive to gametogenesis stimuli. However, in female gametocytes, PfMiGS failed to co-localize with the OB-marker Pfg377 and this did not allow us to determine whether these punctate structures are OB-like vesicles.

Overall our results indicated a large overlap between the secretomes of *P. berghei* and *P. falciparum* gametocytes even though the latter species seems to display a less strict gender specificity. In conclusion, the present study expands our knowledge of the *P. falciparum* gametocyte secretory organelles and gives a preliminary characterization of a set of markers responding to gametogenesis stimuli, making them interesting candidates for future functional studies to investigate their potential role in gamete emergence.

## Supplementary Information

Supplementary Information 1.

Supplementary Information 2.

Supplementary Information 3.

## Data Availability

Mass spectra files are available at ftp://massive.ucsd.edu/MSV; proteomic datasets, supporting the conclusions of this article, are available in supplemental Tables1 and 2.

## Abbreviations

OBs: Osmiophilic bodies
PVM: Parasitophorous vacuole membrane
MOBs: Male osmiophilic bodies
LC: Liquid chromatography
RPLC: Reversed-phase liquid chromatography
FDR: False discovery rate
IFA: Immuno-fluorescence analysis
DAPI: 4′,6-Diamidino-2-phenylindole
GEST: Gamete egress and sporozoite traversal
DPAP2: DiPeptidyl AminoPeptidase 2
SUB2: Subtilisin-like protease 2
MDV1: Male development gene 1
PLD: Putative lactate dehydrogenase
PSOP1: Putative secreted ookinete protein 1
PMX: Plasmepsin X
DPA: Putative deoxyribose-phosphate aldolase
MiGS: The microgamete surface protein
