# Supplementary material for: Identification and preliminary characterization of Plasmodium falciparum proteins secreted upon gamete formation
Source: Sci Rep. 2022 Jun 10;12:9592. doi: 10.1038/s41598-022-13415-7 (PMC9187623; doi:10.1038/s41598-022-13415-7)
Supplement: Supplementary file 1 — Supplementary Information 1. [file 41598_2022_13415_MOESM1_ESM.docx]

**Supplementary figure legends**

**
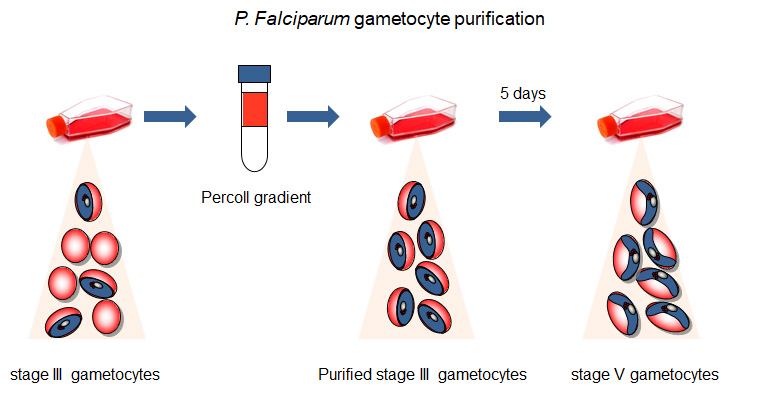
**

**Fig. S1. *P. falciparum* gametocyte purification**. Schematic of the procedure used to produce and purify synchronous mature gametocytes. Cultured *P. falciparum* 3D7 line, was induced to produce gametocytes by starvation and asexual stages were killed 24 hours after induction by 0.05 M N-acetyl glucosamine treatment, to obtain synchronous gametocytes. At day five post induction, *P. falciparum* stage III gametocytes were purified by 60% Percoll gradient to remove uninfected erythrocytes and put back into culture for additional 5 days to obtain mature stage V gametocytes.

**
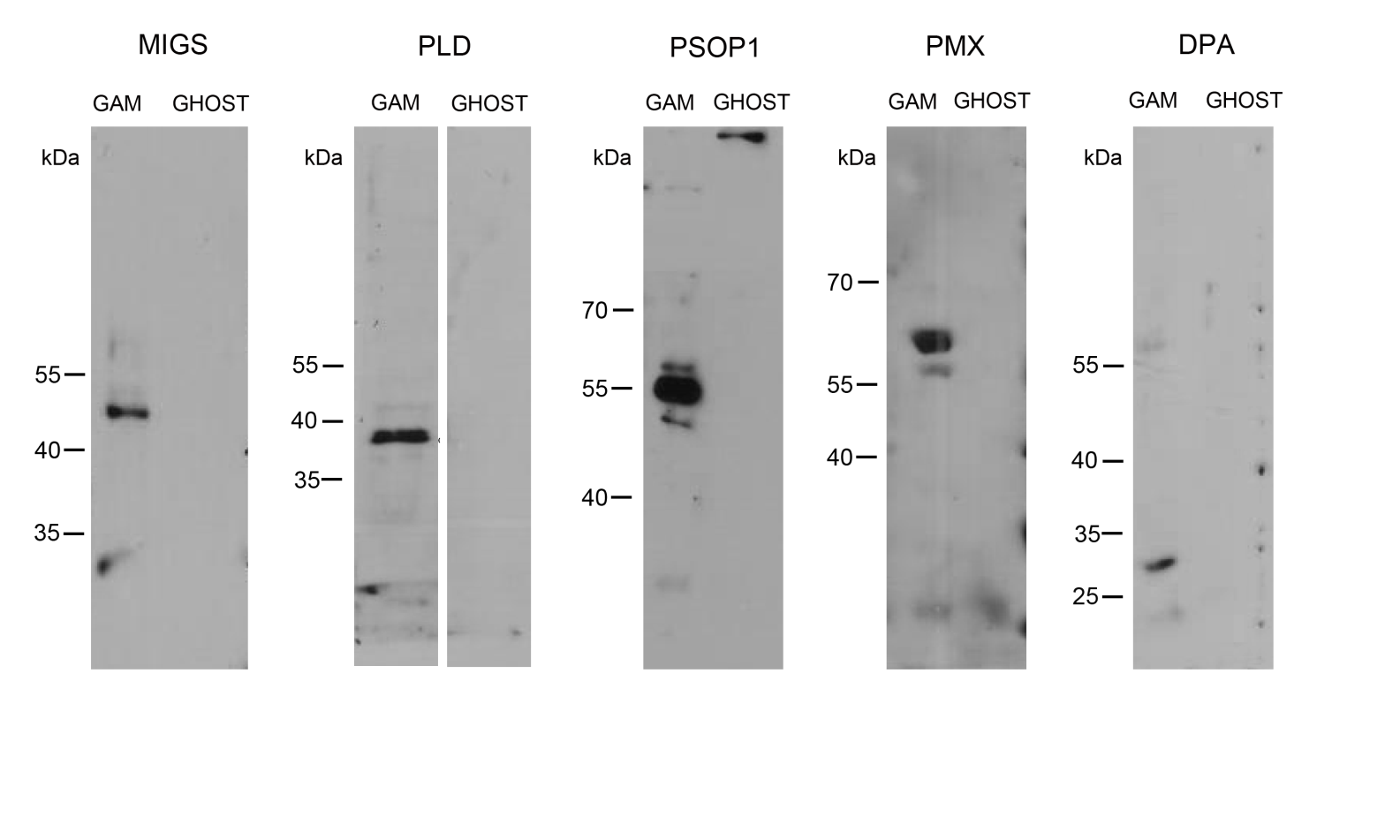
**

**Fig. S2.** **Antibody validation**. Protein extracts from *P. falciparum* purified stage V gametocytes and uninfected erythrocyte ghosts, used as negative controls, were gel-separated and transferred to a nitrocellulose membrane. Primary antibodies were used at a 1:1000 dilution. Expected molecular weights: MIGS 45 kDa; PLD 37 kDa; PSOP1 53 kDa; PMX 65 kDa; DPA 29 kDa.


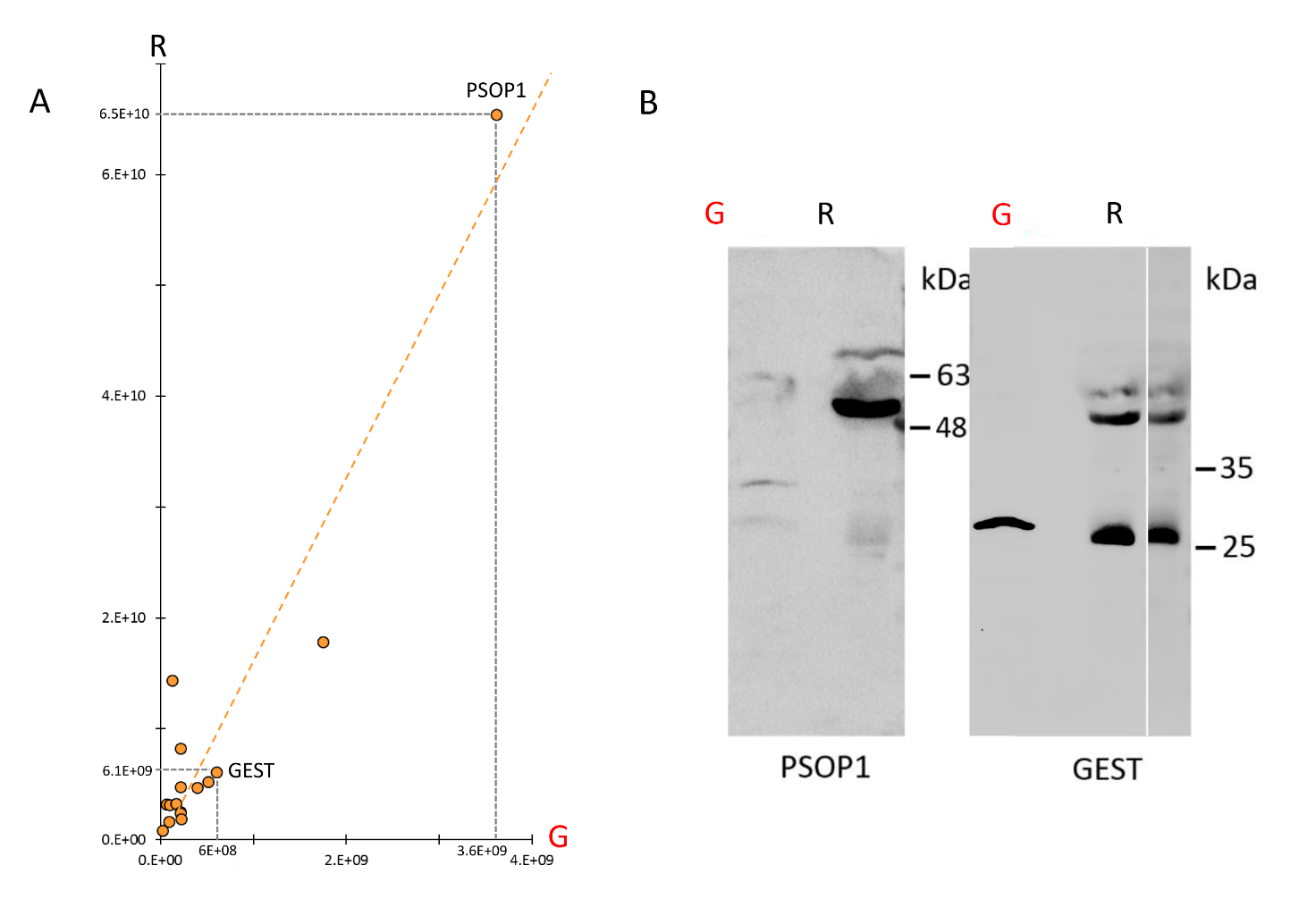


**Figure S3.** **Comparison of protein abundances.** A) Abundances of overrepresented proteins released during gametogenesis (R) plotted vs abundances of total non-induced gametocytes (G). Labels indicate the position of PSOP1 and GEST for which the antibody validation (panel B) confirms an increase in protein amount in egress supernatant.

**A**


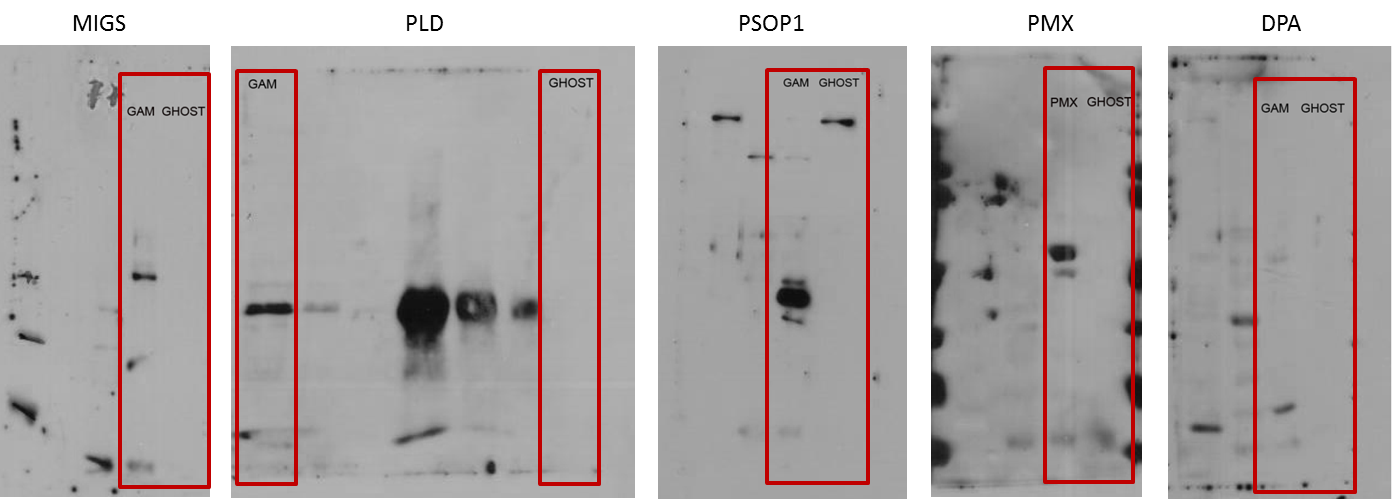


**B**


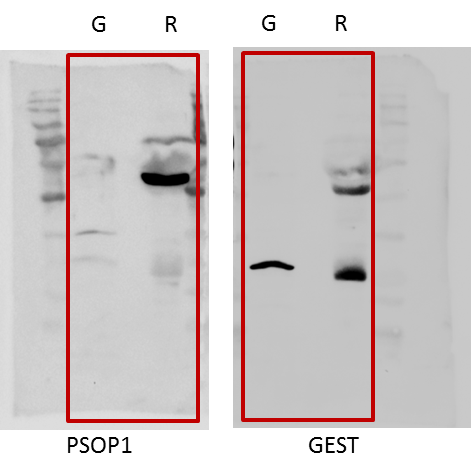


**Figure S4. Full-length gel images.** Western blots utilized to arrange Fig. S2 (A) and Fig. S3 (B). Cropped lanes are boxed in red.

**Table S3.** **Colocalization analysis**. Double IFAs were performed on *P. falciparum* gametocytes using mouse antibodies against the four selected candidates (MIGS, DPA, PMX, PSOP1 and PLD) and rabbit antibodies against the OB marker PfG377. The percentage of overlap between red and green fluorescence was calculated using ImageJ software. The mean value obtained by measuring four independent images and the standard deviations (st.dev) are indicated.
